# Systemic effects of epidural methylprednisolone injection on glucose tolerance in diabetic patients

**DOI:** 10.1186/1756-0500-4-552

**Published:** 2011-12-21

**Authors:** Pascal Zufferey, Charly Bulliard, Gerald Gremion, Marial Saugy, Alexander So

**Affiliations:** 1 DAL (Département de l'appareil locomoteur), CHUV (Centre hospitalier universitaire vaudois), Lausanne, Switzerland; 2 HIB (Hôpital inter-cantonal de la Broye), Estavayer-Le-Lac, Lausanne, Switzerland; 3 Swiss Laboratory for Doping Analyses, University Center of Legal Medecine, Geneva, Lausanne, Switzerland; 4 Centre Hospitalier Universitaire Vaudois and University of Lausanne, Ch. des Croisettes 22, 1066, Epalinges Lausanne, Switzerland

## Abstract

**Background:**

Several studies have shown that in diabetic patients, the glycemic profile was disturbed after intra-articular injection of corticosteroids. Little is known about the impact of epidural injection in such patients. The goal of this study was double, at first comparing the glycaemic profile in diabetic patients after a unique injection of 80 mg of acetate methylprednisolone either intra-articular or epidural and secondly to compare the amount of systemic diffusion of the drug after both procedures.

**Methods:**

Seventeen patients were included. Glycemic changes were compared in 9 diabetic patients following intra-articular (4 patients) and epidural injections (5 patients).

Epidural injections were performed using the sacral route under fluoroscopic control in patients with lumbar spinal stenosis. Diabetes control had to stable for more than 10 days and the renal function to be preserved. Blood glucose was monitored using a validated continuous measuring device (GMS, Medtronic) the day before and for two days following the injection. Results were expressed in the form of daily glycemic profiles and as by mean, peak and minimal values +/- SD. The urinary excretion of methylprednisolone after the 2 routes of injection was analyzed in 8 patients (4 in each group). Urine samples were cropped one hour before the injections, then 4 times during the first day and 3 times a week for 2 weeks. The measurements included the free and conjugated fraction

**Results:**

The glycaemic profile remains unchanged with no significant changes in the group of the 5 diabetic patients receiving epidural injections.

On the other end, the average peak and and mean values were enhanced up to 3 mmol/l above baseline two days after the infiltration in the groups of the 4 diabetic patients infiltrated intra-articular. The mean urinary excretion of the steroid was about ten times higher in the intra-articular versus epidural group: 7000 ng/ml versus 700 ng/ml. Looking at each individual there were marked differences especially after intra-articular injections.

**Conclusion:**

This is the first study to show that a single epidural steroid injection of 80 mg depot methylprednisolone had no effect on the glycemic control in diabetic patients. The absence of glycemic control changes correlated well with the very low urinary excretion of the drug after epidural injection.

**Trial registration:**

NCT01420497

## Background

Several studies [1-3] have shown that in diabetic patients, the glycaemic profile can be profoundly disturbed after an intra-articular corticosteroid injection. The elevation of blood glucose is attributed to diffusion of the injected drug from the joint into the systemic circulation. The amount of steroids in the systemic compartment and the duration of local and systemic effects are related to the solubility of the preparation, the dose injected and probably the degree of inflammation of the joint [3,4]. Amstrong [5] have shown that plasma steroid levels could vary up to five times from one patient to another following injection into the knee joint. On the contrary, little is known about the kinetics of absorption and systemic diffusion of methylprednisolone or other steroid preparations after epidural injection. Jacob et al. [6] suggested that very little of the drug diffused outside the epidural space since they were not able the measure any significant plasma concentration of the drug after an epidural injection of 80 mg of acetate of methylprednisolone. Moreover, no study has evaluated, to our knowledge, the impact of epidural injection of an depot steroid on the glycaemic profile in diabetic patients.

In a former study [7], we have evaluated systemic diffusion of methylprednisolone, triamcilone and depot bethametasone in 25 patients either after intra articular or intra-muscular injections by measuring the urinary excretion of the compounds. The mean excretion after intra-articular was slightly lower than after intra- muscular but in the same range.

Our study had two objectives. Firstly, we wished to compare the effects on the glycaemic profile after an injection of 80 mg of methylprednisolone acetate either by the epidural route or intra-articularly, in diabetic patients. Secondly, we compared the systemic diffusion of steroids in the two procedures.

Due to the small number of patients in each group statistics were limited to Student Test comparison. *P*< 0.05 was considered as significant.

## Methods

The protocole was approved by the ethical committee of the university of Lausanne Switzerland. Seventeen non consecutive patients and healthy subjects were included in the study between September 2008 and May 2010. All gave their informed consent.

Glycemic changes were compared in 9 diabetic patients following intra-articular (4 patients) and epidural injections (5 patients). The urinary excretion of methylprednisolone after the 2 routes of injection was analyzed in 8 patients (4 in each group). The characteristics of the patients and the participants are summarized in Table 1.

**Table 1:** Characteristics of the participants

	Intra-articular injected		Epidural injected	
	
	Glycaemic evaluation	Urinary excretion evaluation	Glycaemic evaluation	Urinary excretion evaluation
Nbr analyzed	4	4	5	4

Mean age [years] (SD)	56(6)	50 (4)	65 (9)	61(6)

Sex[M/F]	3/2	2/2	3/2	2/2

Diabetic patients	4	1	5	2

Insulin treated	3	1	4	2

Mean BMI(SD)	27(6)		31(10)	

Duration of the illness[years]	4		5	

None of the patients had a renal impairment which could interfere with renal elimination of the steroids. The characteristics of Diabetes (duration of the illness type of treatment were similar in both groups) Fasting glycaemia had to be stable for at least 10 days before steroid injection and no changes in diabetic treatment or in life-style were permitted during the three days of the study.

Blood glucose was measured using a continuous device (CGMS, Medtronic, Switzerland) the day before [day 1] and for two days following the injection. The CGMS sensor was inserted under the abdominal skin. For the three days, the glycaemic profile was recorded every 5 min, and the data stored on a portable device attached to a belt. At the end of the evaluation, the data were transferred to a computer for analysis. The device permitted the test subject to take part in normal daily activities. The accuracy and reproducibility of the measurements taken by the CGMS sensor have been confirmed [8]. Results were expressed in the form of daily glycaemic profiles as well as by mean, peak and minimal values +/- SD.

Eighty mg of acetate methylprednisolone [Depomedrol^® ^MSD] was administered by either the intra-articular route or by epidural injection. The same experienced rheumatologist who was not in charge of the diabetes made all the infiltrations. Epidural injections were performed using the sacral route under fluoroscopic control with prior injection of contrast agent in order to confirm the exact localisation of the product [9]. The indications for steroid injections were symptomatic spinal stenosis in all the patients. Intra-articular injections were performed in patients with either an inflammatory (3 times) or degenerative diseases (3 times) in the knee (6 times) according to standard procedure and in the shoulder under ultrasound guidance (2 times).

For urinary excretion measurements, urine samples were collected one hour before the injection, then 4 times during the first day and finally 3 times a week for 2 weeks each two days, between 7-9 h AM the morning. The measurements included the free and the conjugated fraction of the injected steroid and were all performed by the Swiss Laboratory for Doping Analyses according to a validated and published method [7,10].

## Results

**Figure 1:**
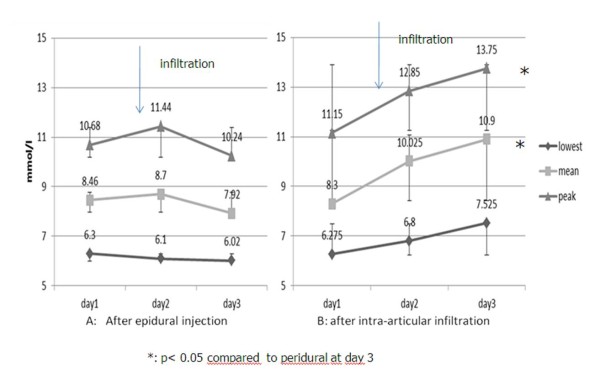
**Mean peak, mean and lowest blood glucose values+/_SD evolution before and after the infiltrations**.

**Figure 2:**
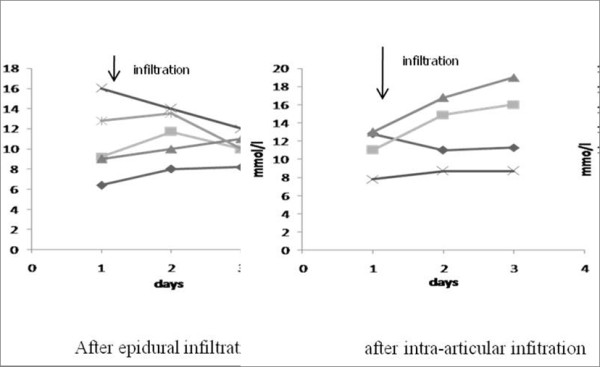
**Individual peak blood glucose evolutions before and after the infiltrations**.

At day 0, the mean blood glucose values were similar before steroid injections in the two groups. In the group of diabetic patients who received epidural injections, the glycaemic profile remained unchanged with no significant changes and very few individual differences (Figures 1 and 2). In contrast, the group of diabetic patients who received intra-articular injections showed mean elevation of peak blood sugar levels of 3 mmol/l two days after the infiltration (at day 3). These changes remain however not significant compared to baseline values due to marked individual variations (Figure 1). By cons, the peak, and mean values after the intra-articular were at day 3 significantly higher than after epidural injections (*p*< 0.05) although the baseline values were identical in both groups (Figure 2). No local or systemic complication or side effect following the infiltrations occurred in both groups.

**Figure 3:**
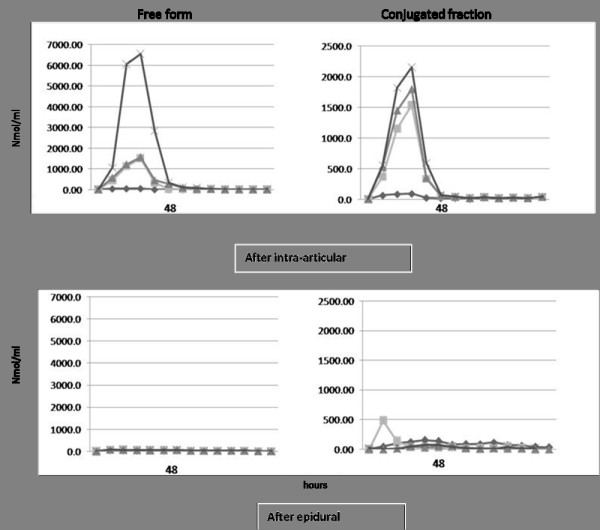
**Urinary excretion rate of methylprednisolone after the infiltrations**.

In the second part, we proceeded to analyse the urinary steroid excretion after each mode of administration. Both conjugated and free forms of methylprednisolone were detected after intra-articular injection, with a peak of excretion during the first 24 first hours after injections. Steroid levels were minimal after 48 h and undetectable after 200 h (Figure 3) in both group. Only some conjugated form of the product could be detected after epidural infiltration.

For this conjugated form, the mean of highest + 3 standard deviations was about ten times higher in the intra-articular versus epidural group: 7000 ng/ml versus 700 ng/ml (Figure 3). We noticed individual differences in the intra-articular group, not linked to the diabetic status. The range of excretion remained for 3 out 4 of them much higher than after epidural infiltration. They were no differences in the epidural group, between the 2 diabetic and the 2 non-diabetic patients. In all of them, the excretion was much lower than after intra-articular infiltrations (Figure 3)

## Discussion and conclusions

To our knowledge, this is the first study to show that a single epidural steroid injection of 80 mg depot methylprednisolone had no effect on the glycaemic profile. Different results were obtained after epidural injection of soluble betamethasone and soluble cortivazol [11,12]. The authors of this study showed a significant increase of glycaemia in the evening following the injection. The glycaemic profile remained also higher for two following days but the increase was no longer significant. This difference between the two studies could be related to the type of steroid used [soluble versus depot steroid] which have been shown to have different metabolism and speediness of elimination in human and in experimental animal models [13].

On the contrary, intra-articular methylprednisolone at the same dose induced a trend to increases of blood glucose in the two days following intra-articular injection. Identical observations have been reported with the same and other depot steroids [1-3]. In most studies, the peak of glycaemia was reached within 48 h and declined rapidly afterwards [2,3]. This could not be confirmed since, in our study, the observation was limited to 2 days after the injections. Most authors mentioned great individual variations as in our work. The small number of patients included in our study does not permit us to determine the causes of such differences. It could be due to the location of the joint infiltrated [knee, shoulder] and the type of arthropathy [inflammatory versus degenerative one].

The absence of blood glucose modification after epidural injection correlated well with the very low urinary excretion of the drug. Indeed the mean excretion was about ten times less than that observed in our patients after intra-articular infiltration and in a previous study [7] after intra-articular or intra muscular injections. These results suggest that following epidural injections, methylprednisolone remains mostly locally in the epidural space and only small amounts enter the systemic compartment. There is however no data in the literature as how corticosteroids injected in the epidural space are metabolized. The synovial membrane particularly when inflamed probably does not behave like the epidural membrane and the volume of dilution in the epidural space is much larger than in most synovial cavities.

After intra-articular injections, we observed important individual variations of urinary excretion which could in part explain the various glycaemic profiles. These variations cannot be explained by the localisation of the injections since 3 out of 4 patients were infiltrated in to the knee. They have been found with other steroid compounds but anyhow in most patients the urinary excretions remain much higher than the ones observed after epidural infiltrations [9,10].

There are several limitations to this study. First the product used: acetate of methylprednisolone. Although it has been the most intensively studied steroid compound in epidural injections [14] is no longer officially recommended in some countries (Frances, Switzerland). This restriction is however also extended to most depot steroid because of a potential although poorly documented link between injections and rare cases of arachnoiditis. The second is the small number of patients included. However, in the epidural group, the results either for the glycaemic profile or the urinary excretion rate are so convergent that reliable conclusions can reasonably be drawn. Moreover the presence of diabetes does not influence urinary excretion, very low in all the patients. This is not the case in the intra-articular infiltrated groups. Potential causes of the discrepancies between patients in the post-injection glycaemic profile have been mentioned above. The same remarks can be made for urinary excretion rate. Moreover, as the patients infiltrated in the two parts of the study were not strictly identical no individual correlation between glycaemia and urinary excretion is feasible. For all these reason no definite conclusion about the risk of diabetic decompensation after an intra-articular at an individual level can be made.

In conclusion our data have shown that epidural injection with methylprednisolone can be considered as safe in diabetic patients. Further extensive studies are needed to draw similar conclusions after intra-articular injection.

## Competing interests

The authors declare that they have no competing interests.

## Authors' contributions

CB and GG help to design the study and collect the cases, MS help to review the manuscript. All authors read and approved the final manuscript.
